# Targeting and assembly of components of the TOC protein import complex at the chloroplast outer envelope membrane

**DOI:** 10.3389/fpls.2014.00269

**Published:** 2014-06-11

**Authors:** Lynn G. L. Richardson, Yamuna D. Paila, Steven R. Siman, Yi Chen, Matthew D. Smith, Danny J. Schnell

**Affiliations:** ^1^Department of Biochemistry and Molecular Biology, University of Massachusetts, AmherstMA, USA; ^2^Department of Biology, Wilfrid Laurier University, WaterlooON, Canada

**Keywords:** chloroplast, outer envelope membrane, protein targeting, translocon, TOC assembly, protein import

## Abstract

The translocon at the outer envelope membrane of chloroplasts (TOC) initiates the import of thousands of nuclear encoded preproteins required for chloroplast biogenesis and function. The multimeric TOC complex contains two GTP-regulated receptors, Toc34 and Toc159, which recognize the transit peptides of preproteins and initiate protein import through a β–barrel membrane channel, Toc75. Different isoforms of Toc34 and Toc159 assemble with Toc75 to form structurally and functionally diverse translocons, and the composition and levels of TOC translocons is required for the import of specific subsets of coordinately expressed proteins during plant growth and development. Consequently, the proper assembly of the TOC complexes is key to ensuring organelle homeostasis. This review will focus on our current knowledge of the targeting and assembly of TOC components to form functional translocons at the outer membrane. Our analyses reveal that the targeting of TOC components involves elements common to the targeting of other outer membrane proteins, but also include unique features that appear to have evolved to specifically facilitate assembly of the import apparatus.

## Introduction

The plastids constitute a diverse array of organelles, which play central roles in plant growth, development, and defense by providing a remarkable range of metabolic and physiological capabilities in different cell and tissue types (Lopez-Juez and Pyke, 2005; Rolland et al., 2012; Jarvis and Lopez-Juez, 2013). The differentiation and maintenance of plastids rely on a complex interplay between plastid and nuclear genomes, requiring coordinate changes in the expression of nucleus- and plastid-encoded genes (Jarvis and Lopez-Juez, 2013). These events trigger modest changes in subsets of plastid proteins, such as the increase in chaperone expression in response to abiotic stress (Taylor et al., 2009), or result in near complete remodeling of the organelle proteome as occurs when non-green etioplasts undergo extensive biochemical and morphological changes to form chloroplasts during photomorphogenesis (Kami et al., 2010). Although these biogenetic events are initiated at the level of transcription, they are ultimately reliant upon the selective import of subsets of several thousand nucleus-encoded proteins into the organelles after synthesis in the cytoplasm (Li and Chiu, 2010; Jarvis and Lopez-Juez, 2013; Shi and Theg, 2013).

A multimeric complex in the plastid outer envelope membrane, referred to as TOC (translocon at the outer membrane of chloroplasts), recognizes the majority of plastid-destined proteins at the organelle surface (Kessler and Schnell, 2009; Chang et al., 2012). TOC components bind the N-terminal transit peptides of newly synthesized preproteins and function in coordination with a second complex at the inner envelope membrane, referred to as TIC (translocon at the inner membrane of chloroplasts), to provide direct transport of preproteins from the cytoplasm to the stroma (Li and Chiu, 2010; Jarvis and Lopez-Juez, 2013). Plants express multiple isoforms of differentially expressed TOC complexes, each of which appears to preferentially mediate the import of subsets of proteins (Jarvis et al., 1998; Bauer et al., 2000; Kubis et al., 2003; Ivanova et al., 2004; Kessler and Schnell, 2009; Inoue et al., 2010; Bischof et al., 2011; Infanger et al., 2011). Both the levels and variety of different TOC complexes appear to be critical for maintaining organelle homeostasis during developmental and physiological changes (Jarvis et al., 1998; Bauer et al., 2000; Kubis et al., 2003; Ivanova et al., 2004; Kessler and Schnell, 2009; Inoue et al., 2010).

The dynamic role of TOC complexes in the recognition and discrimination of plastid-destined preproteins places the TOC machinery at a key hub in protein import and plastid biogenesis. The proper targeting and dynamic assembly of translocon components is key to ensuring that the capacity for protein import and the complement of functionally distinct translocons adjust in coordination with changes in gene expression. This review will focus on examining our current knowledge of the targeting and assembly of TOC components to form functional translocons at the outer membrane. Specifically, we will address aspects of TOC component targeting that conform to general principles of outer membrane targeting, as well as those features that are unique to TOC components that might have evolved specifically to facilitate assembly of the translocons. Previous reviews have included discussions of the mechanism of targeting of individual TOC components to the outer membrane (Li and Chiu, 2010; Kim and Hwang, 2013; Shi and Theg, 2013). We will include these studies, but with the intent of developing models for how the targeting of individual TOC components are coupled to the assembly of functionally diverse translocons that are key contributors to plastid biogenesis.

## Overview of TOC function

**Figure 1:**
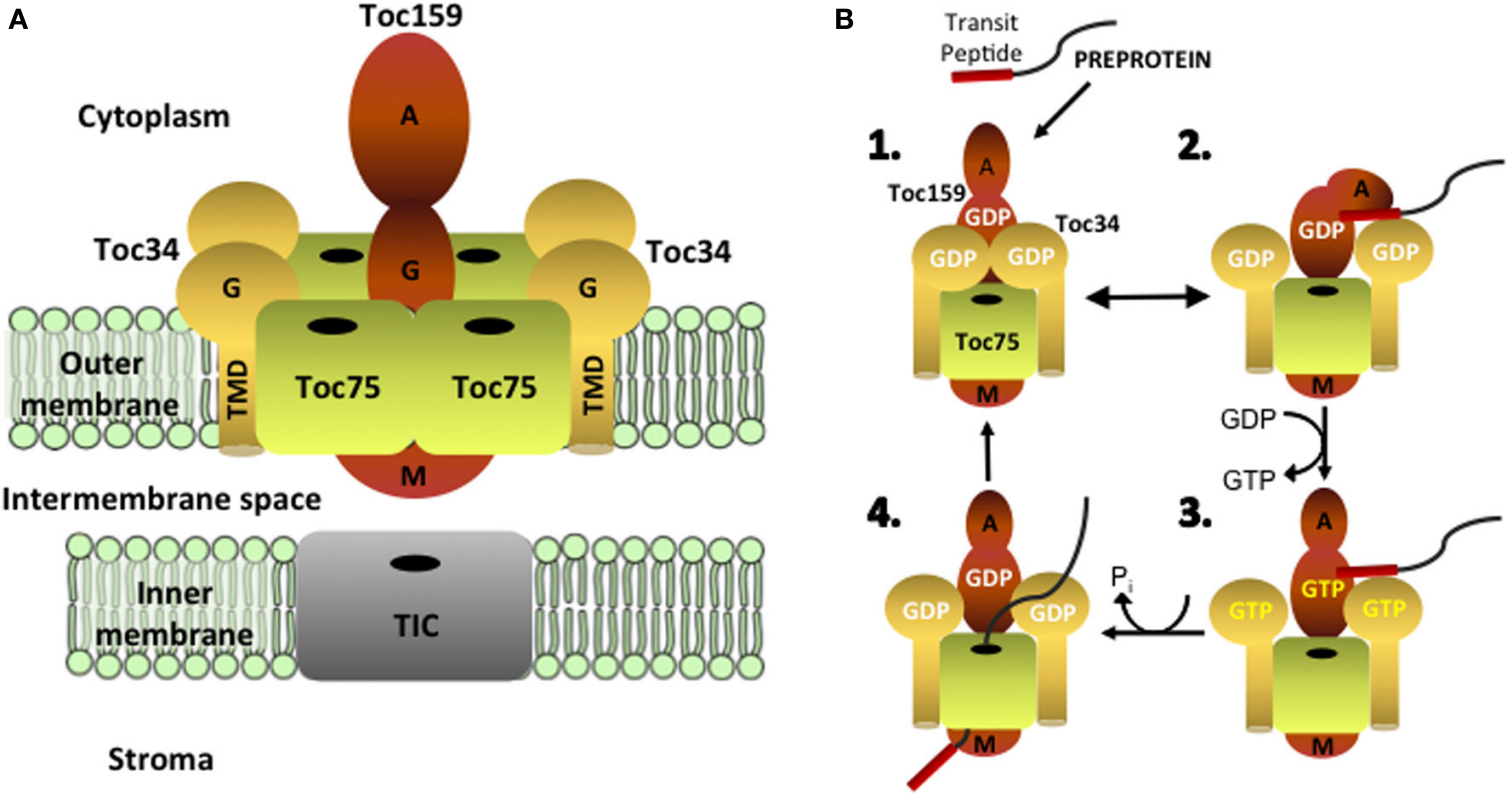
**Structural organization and function of the TOC complex. (A)** The TOC complex consists of two integral membrane GTPases, Toc159 and Toc34, which assemble with the protein-conducting channel, Toc75, in a stoichiometry estimated at 4:4:1 or 3:3:1 (Toc75:Toc34:Toc159). Toc159 has a tripartite structure, consisting of cytoplasmically exposed acidic (A) and GTPase (G) domains and a C-terminal membrane anchor (M) domain. Toc34 contains a cytoplasmic GTPase (G) domain and a single α-helical transmembrane domain (TMD). Toc75 is a β–barrel membrane protein with a predicted 16 membrane-spanning β–strands. **(B)** Protein import into chloroplasts is initiated by the binding of the transit peptide of nuclear encoded preproteins to the G-domains of Toc159 and Toc34 (step 1). Binding of the transit peptide at the G-domains of the receptors triggers changes in receptor dimerization (step 2), which allow for GDP/GTP exchange (step 3). GTP hydrolysis results in the transfer of the preprotein into the Toc75 channel and the initiation of membrane translocation (step 4).

The TOC machinery consists of a core complex containing two related GTP-dependent preprotein receptors, Toc34 and Toc159, which stably interact with a membrane channel, Toc75 (Figure 1A). Toc75, Toc34, and Toc159 are integral membrane proteins that form complexes in the outer membrane with a minimal size of 800 kDa and a stoichiometry estimated at 4:4:1 or 3:3:1 (Toc75:Toc34:Toc159) (Schleiff et al., 2003; Kikuchi et al., 2006; Chen and Li, 2007). Toc34 and Toc159 bind to the transit peptides of newly synthesized preproteins at the chloroplast surface via their GTPase domains (G-domains) and initiate translocation across the outer membrane by transferring preproteins to Toc75 through a series of intermolecular events controlled by their intrinsic GTPase activities (Figure 1B) (Kessler and Schnell, 2002; Li et al., 2007; Chang et al., 2012; Lee et al., 2013). Genetic and biochemical data indicate that transit peptide binding at the receptors regulates both homo- and heterodimerization between their cytoplasmic GTPase-domains, which in turn controls nucleotide exchange, hydrolysis and the initiation of preprotein translocation (Bauer et al., 2002; Smith et al., 2002; Jelic et al., 2003; Weibel et al., 2003; Becker et al., 2004; Yeh et al., 2007; Koenig et al., 2008a,b; Lee et al., 2009; Rahim et al., 2009; Oreb et al., 2011). Biochemical studies using pea chloroplasts and genetic studies in Arabidopsis demonstrate an essential role for Toc75 in plastid protein import (Perry and Keegstra, 1994; Schnell et al., 1994; Tranel et al., 1995; Ma et al., 1996; Baldwin et al., 2005; Hust and Gutensohn, 2006). Furthermore, structural and electrophysiological studies on reconstituted Toc75 demonstrate that the protein forms a cation-selective β-barrel channel, which is regulated by specific interactions with nuclear encoded preproteins (Hinnah et al., 1997, 2002).

The TOC GTPase receptors are encoded by multi-gene families in vascular plants, and different Toc159 and Toc34 family members assemble in combination with Toc75 to form structurally and functionally distinct core complexes (Jarvis et al., 1998; Kubis et al., 2003, 2004; Ivanova et al., 2004; Kessler and Schnell, 2009; Inoue et al., 2010; Bischof et al., 2011; Infanger et al., 2011; Yan et al., 2014). In Arabidopsis, the Toc159 family includes atToc132, atToc120 and atToc90, in addition to atToc159; and the Toc34 family includes atToc34 and atToc33. Biochemical studies demonstrate that different Toc GTPase receptors confer distinct preprotein selectivities on the core TOC complex (Jelic et al., 2003; Becker et al., 2004; Smith et al., 2004; Inoue et al., 2010). In addition, genetic studies coupled with transcriptomic and proteomic analyses suggest that the substrate preferences of different TOC complexes correspond to specific sets of chloroplast proteins whose expression is coordinately regulated in response to physiological or developmental changes (Jarvis et al., 1998; Kubis et al., 2003, 2004; Ivanova et al., 2004; Inoue et al., 2010; Bischof et al., 2011; Infanger et al., 2011). Collectively, these observations have led to the hypothesis that TOC complexes with different but overlapping substrate specificities are required to ensure balanced and efficient import of sets of coordinately expressed proteins during plastid biogenesis, differentiation and/or in response to stress.

## Targeting and integration of Toc75

Toc75 is a common element of all TOC complexes and it functions not only in general protein import, but also in the targeting and insertion of the TOC GTPases (Table 1). As such, it plays a central role in both the assembly and function of the translocon. Toc75 is encoded by a single gene in all plants examined (Inoue and Keegstra, 2003), and it belongs to the OMP85/TspB superfamily of β–barrel integral membrane proteins (Baldwin et al., 2005; Patel et al., 2008; Hsu and Inoue, 2009; Inoue, 2011; Schleiff et al., 2011). The members of this family are exclusively localized in the outer membranes of Gram-negative bacteria, mitochondria and plastids (Voulhoux and Tommassen, 2004; Schleiff et al., 2011), where they are proposed to play diverse roles in membrane transport and biogenesis. The most extensively studied examples include the bacterial BamA (β-barrel Assembly Machinery protein A) and mitochondrial Sam50 (Sorting and Assembly Machinery 50 kDa) proteins, which function in the integration of β-barrel proteins into the outer membrane in Gram-negative bacteria and mitochondria, respectively (Voulhoux et al., 2003; Gentle et al., 2004; Stroud et al., 2011). Mature Toc75 contains structural features characteristic of OMP85/TspB superfamily members, such as FhaC and BamA (Sanchez-Pulido et al., 2003; Gentle et al., 2005; Clantin et al., 2007; Noinaj et al., 2013), including an N-terminal ~30 kDa region consisting of three repeats of POTRA (POlypeptide-TRansport Associated) domains that extend into the soluble space, and a ~45 kDa C-terminal region constituting the membrane-integrated β-barrel.

**Table 1:** **Summary of the targeting requirements of the core TOC components**.

**TOC component**	**Targeting and integration signals**		**Outer envelope receptor/Protein components**	**Cytosolic factors**	**Energy requirements**	**References**
Toc75	Cleavable, N-terminal, bipartite signal^a,b,c^	Targeting		Unknown	ATP^a^	^a^Tranel et al., 1995
		Transit peptide^a,b^	TOC translocon^a,b,c^			^b^Tranel and Keegstra, 1996
		Membrane Integration				^c^Inoue and Keegstra, 2003
		Poly-glycine region^c^	β-barrel translocase (?) (OEP80^d^?)			^d^Schleiff and Soll, 2005
Toc34	GTPase domain ^e,f^		Proteinaceous components^e,h^	AKR2^k^	ATP^h^	^e^Chen and Schnell, 1997
	Transmembrane domain and C-terminal tail ^e,g^			Hsp17.8^l^	GTP/GDP^e,f^	^f^Qbadou et al., 2003
			Toc34^g^			^g^Dhanoa et al., 2010
			Toc75^i^			^h^Tsai et al., 1999
						^i^Kim and Hwang, 2013
						^k^Bae et al., 2008
						^l^Kim et al., 2011
Toc159	M-domain ^m,n,p^		Toc34, Toc75^t^	Unknown	GTP/GDP^q,r^	^m^Muckel and Soll, 1996
	GTPase domain ^q,r,s^					^n^Lee et al., 2003
						^p^Lung and Chuong, 2012
						^q^Hiltbrunner et al., 2001
						^r^Bauer et al., 2002
						^s^Smith et al., 2002
						^t^Wallas et al., 2003

**Figure 2:**
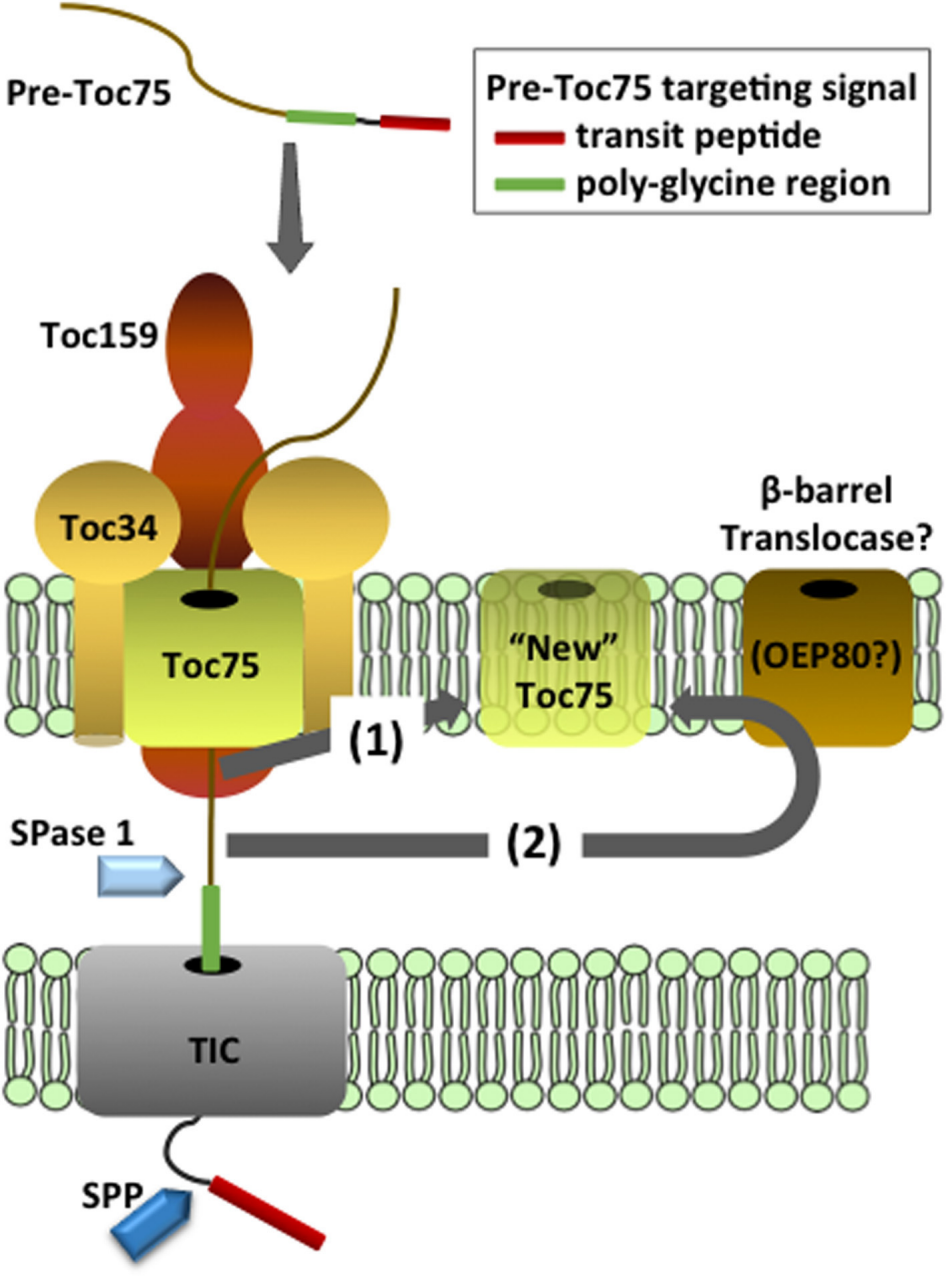
**Models for the targeting and insertion of Toc75**. Pre-Toc75 is synthesized with a bipartite targeting signal containing an N-terminal transit peptide followed in tandem by a poly-glycine region. The transit peptide targets pre-Toc75 to the TOC complex and initiates membrane translocation through the TOC and TIC channels. The stromal processing peptidase cleaves the transit peptide to generate an intermediate form (iToc75), and the poly-glycine region halts complete translocation of iToc75 in the intermembrane space. Two hypotheses have been proposed for the insertion of iToc75 into the outer membrane. Pathway (1) proposes that insertion is mediated directly by the TOC complex. Pathway (2) proposes that iToc75 is engaged by a β–barrel translocase of unknown composition, which catalyzes membrane insertion from the intermembrane space. OEP80 has been proposed as a core constituent of the β–barrel translocase. iToc75 is processed by a type I signal peptidase (SPase 1) to yield mature Toc75 during or shortly after insertion in the outer membrane.

Toc75 is translated as an ~89 kDa precursor (pre-Toc75) and appears to be unique among chloroplast outer envelope membrane proteins (OEPs) in being targeted to the membrane *via* a cleavable N-terminal bipartite targeting signal (Table 1 and Figure 2) (Tranel and Keegstra, 1996). The N-terminal region of pre-Toc75 can target chimeric fusion proteins to the chloroplast stroma, consistent with its function as a canonical transit peptide (Tranel and Keegstra, 1996). The targeting of Toc75 requires ATP and can be competed by the presence of other chloroplast preproteins during *in vitro* import, demonstrating that it employs the TOC translocon for membrane localization (Inoue et al., 2001). A glycine-rich region follows the transit peptide, and functions in the integration of Toc75 into the outer membrane (Tranel and Keegstra, 1996; Baldwin and Inoue, 2006). Pre-Toc75 is processed sequentially; once at amino acid 36, leading to an intermediate form (iToc75; ~85.9 kDa), and again at amino acid 132 resulting in mature Toc75 (mToc75; 75 kDa). Pre-Toc75 is cleaved by the stromal processing peptidase (SPP), indicating that the N-terminus of the precursor reaches the stroma before being sorted to the outer membrane (Tranel and Keegstra, 1996). iToc75 does not reach the stroma and is arrested in the intermembrane space between the outer and inner envelope where it is cleaved by a type I signal peptidase (SPase 1), resulting in mature, functional Toc75 (Inoue and Keegstra, 2003; Inoue et al., 2005; Shipman and Inoue, 2009). Disruption of the gene encoding plastidic SPase I (Plsp1) results in the accumulation of immature forms of Toc75, a severe reduction of plastid internal membrane development, and a seedling lethal phenotype (Inoue et al., 2005; Shipman-Roston et al., 2010).

The glycine-rich region in iToc75 appears to be critical for arresting the import of the protein in the intermembrane space and triggering integration into the outer membrane (Inoue and Keegstra, 2003). It is unclear how the glycine-rich stretch prevents iToc75 from completing translocation across the inner envelope membrane via the TIC translocon, but two hypotheses have been proposed. Proteinaceous components in the intermembrane space or at the inner membrane bind to this region and hold the protein at the outer envelope membrane, or this region could prevent iToc75 from interacting with components that normally direct preproteins through the TIC system (Inoue and Keegstra, 2003; Baldwin and Inoue, 2006). Poly-glycine regions are not bound by DnaK, the bacterial molecular chaperone of the Hsp70 family (Okamoto et al., 2002), and it has been proposed that the iToc75 poly-glycine domain could function similarly to avoid molecular chaperones (e.g., Tic22) in the chloroplast intermembrane space that facilitate translocation of other preproteins across the inner membrane (Inoue and Keegstra, 2003; Baldwin and Inoue, 2006).

### Targeting of Toc75 is distinct from other plastid β-barrel proteins

There are several other plastid-localized members of the OMP85/TspB family, but all lack cleavable transit peptides (Reumann et al., 2005; Hsu and Inoue, 2009). Studies with one of these proteins, OEP80 (Outer Envelope protein, 80 kDa), suggest that it uses a TOC-independent pathway for localization to the outer membrane (Inoue and Potter, 2004). Similar to Toc75, OEP80 is predicted to contain three N-terminal POTRA domains and a C-terminal β-barrel (Hsu and Inoue, 2009), and the gene encoding OEP80 (also referred to as AtToc75-V, At5g19620) is essential in *A. thaliana* (Baldwin et al., 2005; Patel et al., 2008; Hsu et al., 2012). However, OEP80 is not detected in isolated oligomeric TOC complexes, indicating that it is not directly involved in TOC translocon function (Eckart et al., 2002). Although targeting of both Toc75 and OEP80 requires ATP hydrolysis, targeting of OEP80 is not competed by the presence of preproteins utilizing the TOC translocon, and processing of OEP80 was not detected in *in vitro* targeting studies using isolated chloroplasts (Inoue and Potter, 2004). Furthermore, deletion analysis *in vivo* demonstrated that the N-terminal ~52 residues of Arabidopsis OEP80 are not required for the targeting, insertion, or functionality of the protein (Patel et al., 2008; Hsu et al., 2012). These studies indicate that the mechanism of pre-Toc75 targeting and insertion involves steps distinct from those of other plastid β-barrel proteins, such as OEP80.

In Gram-negative bacteria and mitochondria, the targeting of β-barrel proteins to outer membranes requires two translocons, one for targeting proteins into the inter-membrane space between the inner and outer membranes and the second for integrating proteins into the outer membrane (Hagan et al., 2011; Ricci and Silhavy, 2012; Wenz et al., 2014). In both cases, the first step is accomplished by the major protein translocation systems that mediate protein export and import in bacteria and mitochondria, respectively. Translocation of the nascent β-barrel precursors across the cytoplasmic membrane in bacteria occurs through the Sec translocon (Hagan et al., 2011; Ricci and Silhavy, 2012). In mitochondria, this step is mediated by import of newly synthesized nucleus-encoded β-barrel proteins from the cytosol through the translocase of the outer mitochondrial membrane (TOM) (Endo and Yamano, 2010; Dukanovic and Rapaport, 2011). The second step of inserting β-barrel precursors into the outer membrane is mediated by the β-barrel assembly machine (BAM) in bacteria (Tommassen, 2010; Hagan et al., 2011) and the sorting and assembly machinery (SAM) in mitochondria (Endo and Yamano, 2010; Dukanovic and Rapaport, 2011). BAM and SAM are evolutionarily conserved systems containing β-barrel channels, BamA in bacteria and Sam50 in mitochondria, which associate with additional sorting factors to catalyze the targeting and insertion of β-barrel proteins into the lipid bilayer.

Although Toc75, OEP80, BamA, and Sam50 are all members of the OMP85/TspB family, chloroplasts do not appear to possess other components of the β-barrel assembly machinery that are conserved in BAM and SAM complexes of bacteria and mitochondria (Hsu and Inoue, 2009). Consequently, the pathway for β-barrel protein integration at the plastid outer membrane remains to be fully defined. It has been proposed that OEP80 might constitute the core of the β-barrel sorting machinery in chloroplasts (Table 1 and Figure 2) (Schleiff and Soll, 2005; Hsu and Inoue, 2009; Huang et al., 2011). Interestingly, the reduction of OEP80 expression by RNAi in Arabidopsis resulted in the reduced accumulation of Toc75 (Huang et al., 2011). This suggests a role for OEP80 in Toc75 biogenesis, perhaps at a step downstream from initial targeting of pre-Toc75 to the TOC translocon. However, direct evidence for OEP80 participation in β-barrel precursor targeting or insertion is still lacking.

### Models for targeting pre-Toc75 to the outer membrane

Based on existing evidence, several possible mechanisms for pre-Toc75 targeting and integration at the outer membrane have been proposed (Figure 2) (Schleiff and Soll, 2005). Both models include a role for the TOC translocon in the initial stages of pre-Toc75 targeting. The first step involves recognition of the N-terminal transit peptide of pre-Toc75 by the TOC GTPase receptors, followed by translocation into the TOC channel. The observation that the N-terminal transit peptide is processed by the stromal processing peptidase suggests that pre-Toc75 also engages the TIC complex and partially translocates across the inner membrane (Inoue et al., 2001); the glycine-rich segment is proposed to prevent complete translocation of iToc75 in the intermembrane space (Tranel and Keegstra, 1996; Baldwin and Inoue, 2006). At this point, the models diverge. In the first model, the TOC complex directly mediates the insertion of iToc75 into the outer membrane, and membrane integration is coupled directly to protein import (Figure 2). In this scenario, a separate translocase for outer membrane insertion, comparable to the BAM and SAM translocases in bacteria and mitochondria, would not be required. This model is consistent with the observation that Toc75 appears to be unique amongst chloroplast β-barrel proteins in utilizing the TOC-TIC system for targeting (Inoue et al., 2001). Furthermore, protease sensitivity experiments indicate that a significant proportion of iToc75 remains exposed to the cytoplasm during targeting, consistent with an intermediate that remains engaged by the TOC translocon during the sorting and integration process (Inoue et al., 2005).

In the second model, iToc75 would be engaged in the intermembrane space by a second translocase with an activity comparable to BAM or SAM (Figure 2). This translocase would function specifically to integrate β-barrel proteins into the outer membrane. OEP80 is a good candidate for a key component of a chloroplast β-barrel protein translocase, based on its sequence similarity to BamA and Sam50, and the fact that the reduction in OEP80 expression results in reduced accumulation of Toc75 in the outer membrane (Huang et al., 2011). Recent studies in yeast mitochondria demonstrate a close physical association between the TOM import complex and the SAM translocase during β-barrel sorting (Qiu et al., 2013). In a similar scenario, the TOC translocon and an OEP80 β-barrel translocase could cooperate during the sorting of iToc75. A small amount of Toc75 has been shown to immunoprecipitate with OEP80, indicating a potential dynamic interaction between the two proteins (Hsu et al., 2012). If OEP80 does represent a distinct β-barrel protein translocase, it remains to be determined why chloroplasts lack proteins in the intermembrane space similar to the other components of the BAM and SAM translocases that are conserved between Gram-negative bacteria and mitochondria.

The exact role of the transit peptide in Toc75 targeting remains unclear. One possibility is that transit peptide-mediated targeting functions to couple Toc75 sorting with the assembly of new TOC complexes. The transit peptide could ensure that pre-Toc75 remains closely associated with TOC translocons during integration into the outer membrane, regardless of whether or not integration is catalyzed directly by the TOC translocon or by an associated β-barrel translocase. A complex system requiring more than two processing steps for correct insertion of Toc75 would ensure high fidelity targeting of Toc75 to the outer membrane and perhaps facilitate the formation of new translocons (Tranel and Keegstra, 1996). It has recently been proposed that the topology of Toc75 was reversed during evolution resulting in orientation of its N-terminal POTRA domains in the cytoplasm (Sommer et al., 2011). This orientation is the reverse of other known β-barrel proteins in mitochondria and bacteria, and it is possible that the unique targeting pathway is important for determining the unique topology of the protein by orienting the polypeptide in an N_out_ topology during translocation and insertion in the outer membrane.

## Targeting of Toc34

**Figure 3:**
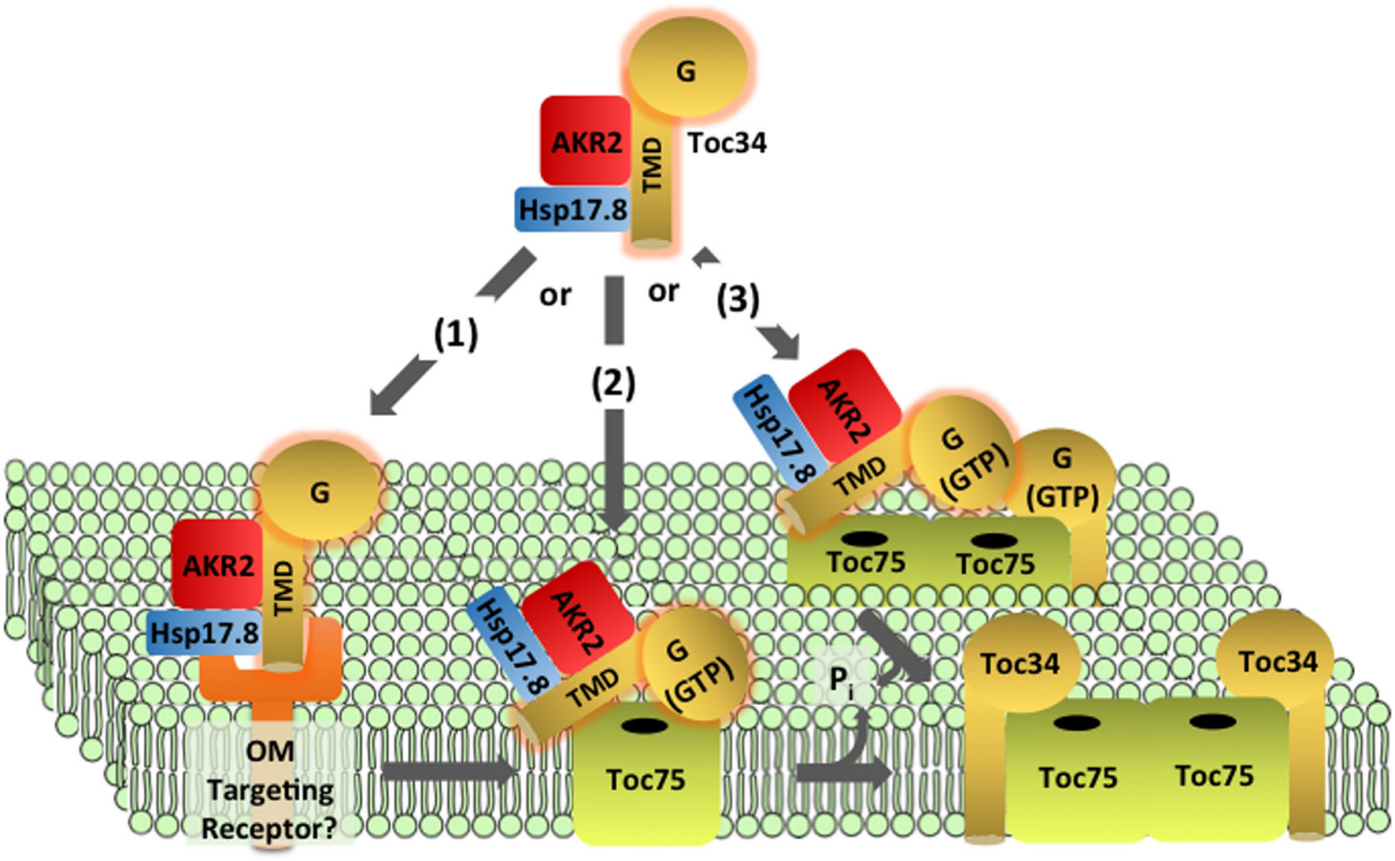
**Targeting and insertion of Toc34 at the outer membrane**. Two cytosolic factors, AKR2 and Hsp17.8, are proposed to recognize the transmembrane domain (TMD) of newly synthesized Toc34 in the cytoplasm and act as molecular chaperones to deliver the protein to the outer membrane. Three hypotheses exist for the recognition and insertion of Toc34 at the outer membrane. In pathway (1), the targeting complex is recognized by an outer membrane targeting receptor(s) and transfers Toc34 to Toc75. Pathway (2) proposes that the Toc34 targeting complex directly binds to Toc75 without the assistance of outer membrane receptors. In pathway (3) binding and insertion of the Toc34 via Toc75 is assisted by interactions of its G-domain with existing Toc34. In all three pathways, Toc75 mediates insertion of the TMD into the membrane and Toc34 stably associates with Toc75.

The Toc34 receptors are encoded by small gene families in many species. All Toc34 isoforms appear to be targeted to the outer membrane by the same mechanism, and therefore we will refer to them collectively as Toc34 and reference specific isoforms only when relevant. Toc34 is anchored in the outer envelope membrane via a C-terminal transmembrane domain (TMD), with its N-terminus (including the G-domain) exposed to the cytosol and a relatively short C-terminal sequence (CTS) oriented toward the intermembrane space (Table 1 and Figure 3) (Li and Chen, 1996, 1997; Gutensohn et al., 2000; Dhanoa et al., 2010). Organellar proteins with this topology are collectively referred to as tail-anchored (TA) proteins (Kim and Hwang, 2013).

Toc34 and other outer membrane proteins with single transmembrane anchors lack a cleavable targeting signal, and the TMD and residues directly adjacent to the TMD are common features of their targeting signals (Lee et al., 2001, 2004, 2011; Hofmann and Theg, 2005; Dhanoa et al., 2010). In addition to the essential role of the TMD, the CTS following the TMD, and interactions with plastid-specific lipids are proposed to play a role in the specific targeting of TA proteins to plastids (Schleiff et al., 2001; Dhanoa et al., 2010). The selective targeting of TA proteins between chloroplasts and other organelles, including mitochondria, peroxisomes and the ER, also involves the degree of hydrophobicity of the TMD (Borgese et al., 2007; Lee et al., 2011), and *in vitro* targeting experiments with isolated organelles suggest that selectivity can occur at the surface of the organelle, independent of cytosolic targeting factors (Kriechbaumer and Abell, 2012).

### Components and energetics of the Toc34 targeting pathway

Toc34 and a second TA protein, OEP9, are recognized by the ankryin repeat cytosolic factor, AKR2, in the cytosol (Table 1 and Figure 3) (Dhanoa et al., 2010). AKR2 appears to participate in the targeting of a variety of plastid, peroxisomal and ER proteins with single, N- or C-terminal TMDs, suggesting that it acts as a general chaperone for TMD-containing proteins by preventing inappropriate interactions during transit from the cytoplasm to boundary membranes (Bae et al., 2008; Dhanoa et al., 2010; Shen et al., 2010; Zhang et al., 2010). Recently a small heat shock protein, sHsp17.8 was identified that mediates specific association of AKR2 with chloroplasts, and enhances targeting of another chloroplast outer membrane protein, OEP7/OEP14 (Kim et al., 2011). It remains to be shown if sHsp17.8 also participates in Toc34 targeting. ARSA1, another cytosolic factor, was implicated in the targeting of Toc34 to chloroplasts in *Chlamydomonas reinhardtii* (Formighieri et al., 2013). ARSA1 is structurally related to the cytosolic targeting factor, GET3/TRC40, which facilitates targeting of TA proteins to the ER in yeast and mammals. *arsa1* mutants appear to selectively impact chloroplast biogenesis and not significantly affect the function of other cellular organelles. There are multiple isoforms of ARSA-like proteins in other plant species, suggesting that one or more ARSA homologs might function in Toc34 targeting in land plants.

The components at the outer membrane that mediate insertion of Toc34 remain to be fully defined. Insertion was initially proposed to be spontaneous (Schleiff and Klosgen, 2001; Jarvis and Robinson, 2004), based on the observation that Toc34 and OEP7/OEP14 were capable of associating with protein-free liposomes in the absence of nucleotide hydrolysis (Qbadou et al., 2003; Wallas et al., 2003; Dhanoa et al., 2010). However, the observations that the insertion of outer membrane proteins is promoted by nucleotide hydrolysis and inhibited by proteolytic treatments of chloroplasts argue for protein-mediated insertion (Hofmann and Theg, 2005). A proteinaceous receptor system specific for the targeting of outer membrane proteins has been proposed, but no components have been identified (Kim and Hwang, 2013). Considerable evidence suggests that Toc75 participates in the insertion of OEP14/OEP7 and similar proteins (Figure 3) (Tu et al., 2004; Hofmann and Theg, 2005). The similarities between Toc34 and OEP7/OEP14 targeting have led to the hypothesis that their targeting pathways share common components, including Toc75 (Figure 3) (Kim and Hwang, 2013).

### Role of the G-domain in Toc34 targeting to TOC complexes

Although similarities exist between the targeting of Toc34 family members and other outer membrane proteins, several aspects of Toc34 membrane integration are unique and likely represent events that facilitate or are required for TOC assembly. For example, the GTPase activity of Toc34 was shown to stimulate its insertion into the outer envelope of isolated chloroplasts (Figure 3) (Chen and Schnell, 1997; Qbadou et al., 2003). Although the mechanism by which GTP-hydrolysis at Toc34 facilitates insertion remains to be investigated, the known interactions of the G-domain with other components of the TOC complex suggest that it may play a role in targeting and/or assembly of Toc34 into TOC complexes. Toc34 dimers interact via their G-domains (Sun et al., 2002), and these homotypic interactions might be involved in targeting of Toc34 to sites of TOC complex assembly (Figure 3). This hypothesis is supported by the observation that insertion of atToc33 and atToc34 is reduced relative to OEP9 in chloroplasts isolated from the atToc33 and atToc34 null mutants, *ppi1* and *ppi3*, respectively (Dhanoa et al., 2010). In an alternative model, Toc34 could interact directly with Toc75 during targeting, and GTPase activity could facilitate insertion and/or stabilization of the interaction with the channel (Figure 3). This model is consistent with the highly stable association between Toc34 and Toc75, reflected in the observation that Toc34 is found exclusively in TOC complexes (Kouranov et al., 1998) in a 1:1 stoichiometry with Toc75. Phosphorylation has been proposed to regulate the association of Toc34 with the translocon and thereby facilitate exchange of components from the complex (Oreb et al., 2008).

## Targeting and membrane integration of Toc159

Members of the Toc159 family function as primary chloroplast preprotein receptors, and play fundamental roles in determining preprotein substrate specificity (Bauer et al., 2000, 2002; Ivanova et al., 2004; Kubis et al., 2004; Smith et al., 2004; Inoue et al., 2010). All Toc159 family members have a unique tripartite structure, consisting of an N-terminal acidic domain (A-domain) and a central GTPase domain (G-domain), both of which are exposed to the cytosol; and a C-terminal membrane anchor domain (M-domain) that is protected from proteolysis and associates with the chloroplast outer envelope membrane through an unknown mechanism (Hirsch et al., 1994; Bauer et al., 2000; Ivanova et al., 2004; Lung and Chuong, 2012). A recent study using a yeast two-hybrid approach demonstrated that the G-domains of Toc159 receptors bind to a wide range of preproteins, and the A-domain alters the relative affinity of each receptor for different classes of preproteins (Dutta et al., 2014). Expression of the M-domain of Toc159 alone can partially complement the seedling lethal phenotype of atToc159 null mutants in Arabidopsis, indicating its central role in formation of the functional translocon (Lee et al., 2003).

The majority of information on the targeting and function of the Toc159 family has been obtained by studying the most abundant isoform in green tissue—atToc159 and psToc159 from Arabidopsis and pea, respectively. For simplicity, we will refer to the family members collectively as Toc159. Evidence to date indicates that the mechanism of Toc159 targeting and insertion involves its G- and M-domains as well as other TOC components, including Toc34 and Toc75 (Wallas et al., 2003).

### Role of the G- and M-domains in Toc159 targeting to TOC complexes

The M-domain encompasses the ~400 most C-terminal residues of the Toc159 protein family (Hirsch et al., 1994; Ivanova et al., 2004; Lung and Chuong, 2012). It is the minimal structural unit to confer protein import capability in Arabidopsis plants lacking full-length Toc159 (i.e., in the atToc159 null mutant, *ppi2*) (Chen et al., 2000; Lee et al., 2003), indicating that integration of this domain is a critical step in TOC complex assembly. Interestingly, while it is known that the M-domain spans the outer membrane and anchors Toc159 in the outer membrane based on its insensitivity to protease treatment and resistance to extraction in isolated chloroplasts, it does not possess any predicted hydrophobic transmembrane domains (Hirsch et al., 1994; Kessler et al., 1994; Muckel and Soll, 1996; Chen et al., 2000). This suggests that the nature of Toc159 membrane association is unique relative to other chloroplast outer envelope proteins.

When fused to an unrelated soluble protein, the M-domain is able to target the fusion protein to the chloroplast surface *in vitro* (Muckel and Soll, 1996), and the M-domain on its own targets to chloroplasts in a transient protoplast expression system (Lee et al., 2003), albeit inefficiently compared to native Toc159. Furthermore, the M-domain binds to isolated chloroplasts and interacts with the Toc34 G-domain *in vitro* (Wallas et al., 2003). These data demonstrate that the M-domain contains intrinsic targeting information for sorting to the outer membrane.

Recently, two Toc159 family members were identified in *Bienertia sinuspersici*, a species that carries out single-cell C4 photosynthesis by the presence of dimorphic chloroplasts in a single chlorenchyma cell (Lung and Chuong, 2012). A bioinformatics analysis of the C-terminal ~100 residues (CTs) of the *B. sinuspersici* receptors, BsToc159 and BsToc132, revealed that this region has chloroplast transit peptide-like properties that are generally conserved in Toc159 homologs from other species (Lung and Chuong, 2012). These features include an overrepresentation of hydroxylated residues, and regions of predicted random coil and amphipathic-helical secondary structure (Lung and Chuong, 2012). Remarkably, this region functions as a transit peptide when fused to the small subunit of Rubisco in reverse orientation to maintain the topology of its interaction with the outer envelope (Lung and Chuong, 2012). These findings raise the intriguing possibility that the C-terminal region facilitates targeting of Toc159 to the outer membrane by mimicking a transit peptide to engage the TOC machinery.

The Toc159 G-domain also appears to contribute significantly to targeting of the receptor, and on its own binds to chloroplasts *in vitro* (Smith et al., 2002), indicating that it possesses intrinsic chloroplast targeting information. A Toc159 mutant deficient in both GTP binding and hydrolysis (Toc159mGTP) fails to complement a Toc159 null mutant (*ppi2*) in Arabidopsis (Bauer et al., 2002). Upon closer examination, it was found that the Toc159mGTP mutant has a reduced efficiency of binding and insertion into isolated chloroplasts (Bauer et al., 2002). The importance of GTPase activity for Toc159 targeting appears to be attributable to nucleotide binding and not hydrolysis itself. This conclusion is supported by the observation that expression of a Toc159 mutant, which binds nucleotide but is defective in GTP hydrolysis, complements the *ppi2* phenotype (Wang et al., 2008). Consistent with this premise, the GDP-bound form of Toc159 is inserted into isolated chloroplasts more efficiently than in its GTP-bound form (Smith et al., 2002). This has led to the hypothesis that nucleotide binding, in particular GDP binding, induces a conformation that renders Toc159 competent for targeting and integration into the outer membrane (Smith et al., 2002).

Toc159 binds to proteoliposomes containing either Toc34 or Toc75; however insertion into the membrane requires both Toc34 and Toc75 (Wallas et al., 2003). A direct role for Toc34 in Toc159 targeting is supported by the observation that Toc159 interacts with Toc34 via their respective G-domains (Hiltbrunner et al., 2001; Bauer et al., 2002; Smith et al., 2002; Wallas et al., 2003; Rahim et al., 2009); an interaction that is regulated by the GTPase activity of both Toc159 and Toc34 (Bauer et al., 2002; Wallas et al., 2003). In addition, when added to *in vitro* targeting assays, soluble Toc34 G-domain can compete for membrane insertion of Toc159 (Hiltbrunner et al., 2001). These data, in conjunction with the role of nucleotide binding on Toc159 targeting, suggest that interactions between the G-domains of the two receptors are important elements in efficient targeting of the receptor to the outer membrane.

Based on the demonstration that the A-domains of Toc159 family members are intrinsically disordered, it has been suggested that they might facilitate the assembly of TOC complexes (Richardson et al., 2009), a function that has been attributed to other intrinsically disordered proteins (Hegyi et al., 2007). The number of proteins with disordered regions positively correlates with the size of macromolecular complexes in yeast and *E. coli*, and it is hypothesized that large unstructured domains allow for simultaneous protein-protein interactions with multiple binding partners, or give flexibility to functional domains within complexes (Hegyi et al., 2007). In this manner, the A-domain of Toc159 could mediate transient interactions between multiple TOC components, either simultaneously or sequentially, during assembly of functional TOC complexes (Richardson et al., 2009). While the possibility is intriguing, definitive evidence for such a role has not yet been reported.

### Working model of Toc159 targeting and insertion

**Figure 4:**
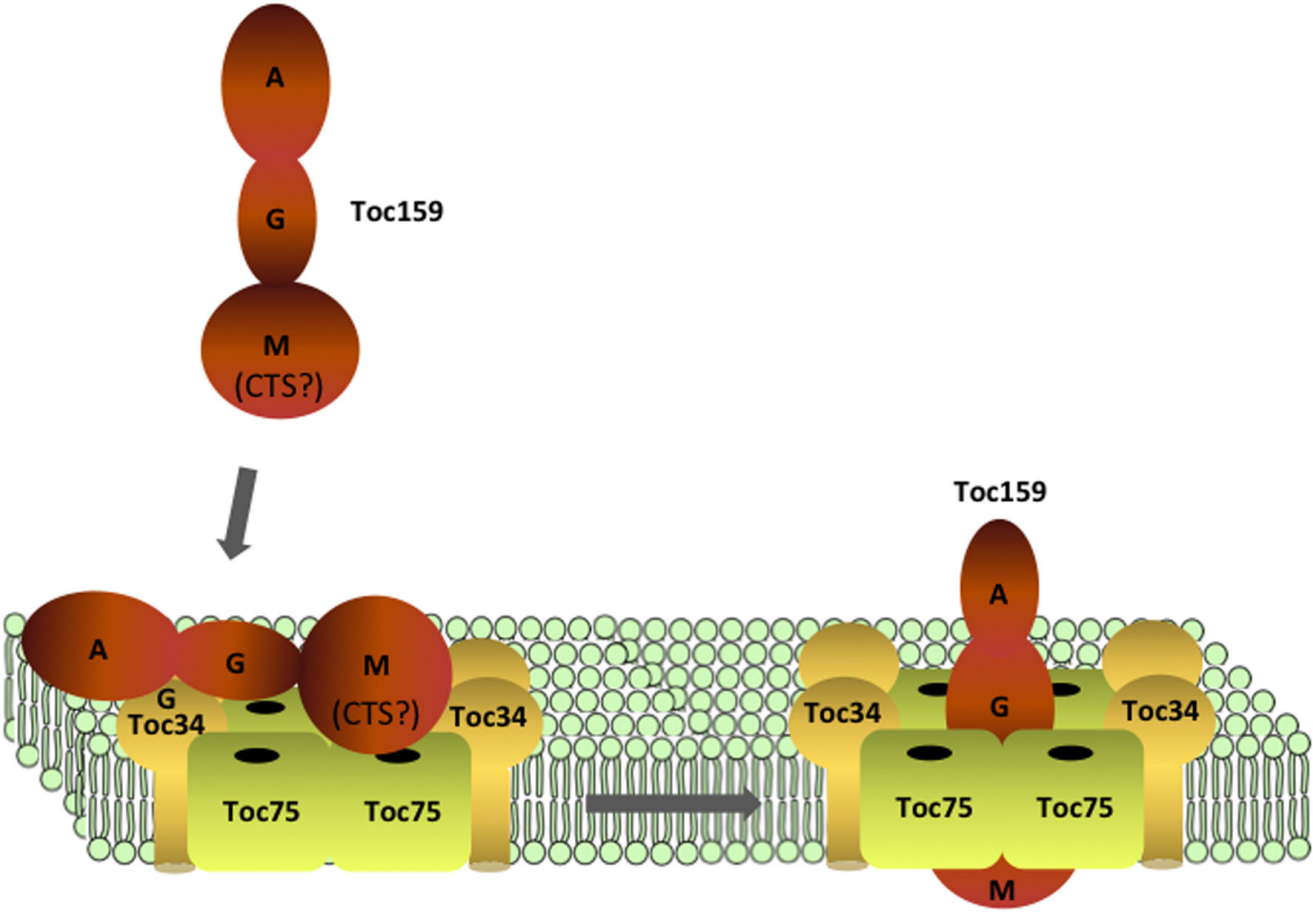
**Targeting and assembly of Toc159 at TOC complexes**. Toc159 is targeted to the outer membrane via interactions between its G- and M-domains with Toc34 and Toc75. Recognition of Toc159 by Toc34 and Toc75 might be assisted by a C-terminal sequence (CTS) within the M-domain, which has properties similar to a transit peptide. Insertion of the M-domain into the membrane is facilitated by nucleotide binding (GDP) at the G-domain, and assembly of Toc159 into TOC complexes results in functional translocons with the ability to import preproteins.

The existing data suggest that the intrinsic targeting information within the G- and M-domains of Toc159 act co-operatively to target the receptor to the chloroplast outer envelope (Figure 4). The targeting and subsequent insertion of the Toc159 M-domain would be facilitated by a nucleotide-dependent interaction between the G-domains of Toc159 and Toc34. Consistent with this model, addition of the Toc159 G-domain *in trans* to isolated chloroplasts stimulated insertion of the M-domain into the outer envelope (Wallas et al., 2003). The M-domain may initially engage the TOC complex through an interaction with Toc34 and/or Toc75. It is intriguing to speculate that the C-terminal region of the Toc159 M-domain takes advantage of the intrinsic transit peptide binding capabilities of Toc34 and Toc75 to specifically target the newly synthesized receptor to nascent TOC complexes (Figure 4) (Lung and Chuong, 2012). In this scenario, the initial targeting of Toc159 to the outer membrane would share elements with the binding of transit peptides at the TOC complex during the import of nuclear encoded preproteins. While the physiological relevance of this transit-peptide-like region to Toc159 targeting remains to be explored in more detail, this unusual targeting mechanism might have evolved to facilitate establishment of the unique membrane association of the M-domain and the interactions of Toc159 with other components of TOC complexes.

It also has been reported that Toc159 exists as a soluble cytoplasmic receptor (Hiltbrunner et al., 2001; Becker et al., 2004; Lung and Chuong, 2012). While this observation may have interesting implications for Toc159 targeting, other data suggest that the soluble form of Toc159 represents a targeting intermediate en route to the chloroplast or a biochemical artifact generated during *in vitro* studies (Becker et al., 2004). Consequently, further studies need to be carried out to unravel the physiological significance of the soluble form and how it might relate to the reported Toc159-actin interaction (Jouhet and Gray, 2009a,b).

## The sequence of assembly of the TOC complex

On the basis of studies investigating the targeting and insertion of individual TOC components, we propose a model for the mechanism of TOC assembly. As described above, evidence supports a role for Toc75 in the targeting and integration of both Toc34 and Toc159 into the outer membrane (Figures 3, 4). We propose that the integration of Toc75 into the outer membrane represents the first step in TOC formation (Figure 2). Pre-existing TOC complexes mediate the targeting of newly synthesized pre-Toc75 to the membrane, indicating that existing TOC translocons play a central role in the formation of new TOC complexes (Tranel and Keegstra, 1996). Full integration of Toc75 at the outer membrane could be mediated by the TOC translocon or in conjunction with a distinct β–barrel assembly machinery that could include OEP80.

Studies in pea demonstrate that the levels of Toc75 significantly exceed those of Toc34 and Toc159 at early stages in development when chloroplast biogenesis and division are maximal, and suggest that up to 50% of Toc75 exists in a form not associated with the GTPases (Kouranov et al., 1998). This would provide a sufficient pool of “free” Toc75 to nucleate the assembly of new TOC complexes. Based on the proposed role of Toc75 in the insertion of outer membrane proteins (Tu et al., 2004; Hofmann and Theg, 2005), we hypothesize that the channel functions in the integration of Toc34 (Figure 3). This hypothesis predicts that Toc75 would interact directly or indirectly with other components of the Toc34 targeting pathway (e.g., AKR2), and the channel formed by Toc75 could provide an interface to facilitate contact between the transmembrane helices of the proteins with the core of the lipid bilayer. Once integrated, Toc34 would remain tightly associated with Toc75.

Toc159 insertion at the outer membrane is dependent upon both Toc75 and Toc34 as demonstrated by reconstitution of the complex from individual components (Figure 4) (Wallas et al., 2003). Therefore, Toc159 is envisioned to be the final addition to the core TOC complex. As discussed above, the M-domain region could take advantage of the transit peptide binding properties of Toc34 and Toc75 to facilitate targeting of Toc159 to newly forming TOC complexes (Lung and Chuong, 2012). The interactions between the G-domains of the Toc34 and Toc159 could provide a recognition component in addition to the M-domain to enhance the efficiency of targeting of Toc159 to the membrane. In this scenario, the interaction of the GTPase domains also could play a role in maintaining the association of Toc159 with the other two TOC components during insertion of the M-domain into the membrane, thereby facilitating the formation of the core complex. The M-domain of Toc159 appears to be the minimal functional component of the receptor (Lee et al., 2003), and therefore the GTPase domains would play a critical role in the efficient formation of functional TOC complexes.

The proposed model, in which Toc75 serves as the nucleating core for the sequential addition of Toc34 and Toc159, also provides a mechanism to couple the targeting and membrane integration of TOC components directly with complex assembly (Figure 4). The interactions that maintain the stability and stoichiometry of the core complex, including functional interactions between the G-domains of the receptors and receptor interactions with Toc75, would be established in concert with targeting of the receptors from the cytoplasm to the membrane. Addition of Toc159 as the final component would complete assembly and serve to “activate” the translocon by integrating the functionally critical Toc159 M-domain into the complex (Figure 4). The model also proposes that Toc34 and Toc159 are the limiting factors in the formation of TOC complexes because of the excess availability of Toc75 in the membrane. Therefore, the levels of expression of the GTPases could determine the rate of formation of new translocons.

## TOC complex assembly in relation to the generation of functionally diverse translocons

The existence of structurally distinct TOC translocons raises interesting questions regarding the mechanisms controlling their formation and abundance. Toc75 is the common element in TOC complexes that contain different combinations of Toc34 and Toc159 family members. The availability of excess Toc75 could provide a pool for rapid assembly of new TOC complexes with newly synthesized Toc34 and Toc159. Although the measurements of the size of TOC translocons are heterogeneous, estimates predict a minimal mass of ~800 kDa, suggesting that each complex contains at least two molecules of Toc159 and six molecules each of Toc34 and Toc75 (Schleiff et al., 2003; Kikuchi et al., 2006; Chen and Li, 2007). Consequently, there is likely to be an active mechanism for the selective assembly of distinct receptors in specific combinations with Toc75. The analysis of TOC translocons in Arabidopsis has shown that two Toc159 family members, atToc159 and atToc132, preferentially assemble with two Toc34 family members, atToc33 and atToc34, respectively (Ivanova et al., 2004). The preferential interactions of the G-domains of the two receptors during targeting and assembly, both homotypic and heterotypic, could result in the assembly of translocons with specific compositions. It is also possible that the divergent A-domains contribute to the assembly of complexes with distinct compositions.

This model is unlikely to fully account for the assembly of distinct translocons. Expression of atToc33 or atToc34 complements the reciprocal Arabidopsis null mutants of either receptor (Jarvis et al., 1998), demonstrating that atToc159 and atToc132 can form distinct, functional translocons when assembled with either atToc33 or atToc34. Furthermore, some vascular plant species appear to only have a single Toc34 gene. For example, rice, a plant with a significantly more complex genome than Arabidopsis, is predicted to contain up to five Toc159 family members, but only one Toc34 ortholog. Therefore, the preferential interactions between Toc159 and Toc34 family members might play a role in the formation of distinct translocons in some, but not all plants species. The specific Toc159 isoform present within the TOC complex appears to be the minimal distinguishing feature of different translocons, consistent with genetic analyses demonstrating that atToc159, atToc132/atToc120 and atToc90 are functionally distinct (Ivanova et al., 2004; Kubis et al., 2004; Infanger et al., 2011). This raises the possibility that each Toc159 family member plays a role in the recruitment of the same or functionally similar isoforms, or exclusion of distinct isoforms into newly forming complexes. Homotypic interactions between the G-domains of Toc159 family members have not been examined in detail, but these interactions could contribute to recruiting the same receptor isoform once an initial Toc159 protein assembles with Toc75 and Toc34. The A-domains of Toc159 receptors play key roles in defining the selectivity of complexes for different import substrates (Agne and Kessler, 2010; Inoue et al., 2010), and it is intriguing to speculate that this domain could also participate in recruiting compatible or excluding incompatible Toc159 isoforms during TOC assembly.

Recent studies provide evidence that the abundance of TOC complexes is controlled by regulated proteolysis via the cytoplasmic ubiquitin-proteasome system (UPS), further highlighting the importance of regulating protein import at the level of the TOC translocon (Ling et al., 2012; Jarvis and Lopez-Juez, 2013). The UPS pathway is proposed not only to participate in the housekeeping turnover of TOC complexes, but also be important in selective degradation of specific TOC isoforms, thereby regulating the proper proportions of different TOC complexes to facilitate changes in the plastid proteome during developmental transitions (Ling et al., 2012; Huang et al., 2013; Jarvis and Lopez-Juez, 2013; Ling and Jarvis, 2013). The UPS mechanism of selective TOC degradation coupled with regulation of TOC assembly would provide an integrated system of controlling the levels of specific translocons in response to physiological or developmental demands.

## Concluding remarks

The translocons mediating the import of nuclear encoded preproteins play central roles in the biogenesis and functional differentiation of plastids. Major attention has been devoted to uncovering the mechanism of preprotein recognition and membrane translocation at TOC and TIC, and it is increasingly clear that the assembly and regulation of these complexes play an important role in organelle function and homeostasis. To date, studies on individual TOC components suggest that their targeting involves elements in common with other outer membrane proteins, but also reveal features that are unique to TOC biogenesis. Detailed studies on the characteristics and relationships of targeting pathways for the TOC proteins, the identification of the components of each pathway, and the definition of the roles of known components are needed to provide a complete picture of the mechanism and regulation of TOC assembly. A more complete picture of targeting and assembly in conjunction with information on the structures and interactions of core TOC components will undoubtedly shed light on key regulatory or quality control checkpoints in the assembly and dynamics of this unique macromolecular assembly. Finally, studies integrating TOC assembly with the newly discovered mechanism of TOC control by regulated proteolysis provides an opportunity to understand how the levels and diversity of the translocons are controlled, and thereby contribute to the plasticity of organelle function in response to developmental and physiological events in the cell.

### Conflict of interest statement

The authors declare that the research was conducted in the absence of any commercial or financial relationships that could be construed as a potential conflict of interest.
